# Colour Doppler and Volume Changes—Anterolateral Thigh Flap: The Sunnybrook Experience

**DOI:** 10.5402/2011/241385

**Published:** 2011-11-03

**Authors:** Antoine Eskander, Danny Enepekides, Kevin Higgins

**Affiliations:** ^1^Department of Otolaryngology-Head and Neck Surgery, University of Toronto, Toronto, ON, Canada M5G 2N2; ^2^Department of Otolaryngology-Head and Neck Surgery, Sunnybrook Health Sciences Centre, Toronto, ON, Canada M4N 3N5

## Abstract

This paper summarizes two recent ALT studies from our institution focusing on the utility of colour flow Doppler's ultrasonography and volumetric changes with radiation. Lastly, we will discuss recommended changes to practice due to the aforementioned studies. Our findings suggest that special care should be made to identify patients at high risk for peripheral arterial disease, and, if the reconstructive surgeon has any concerns, preoperative colour flow Doppler's ultrasonography should be undertaken to better characterize the perforator anatomy and avoid inappropriate flap elevation in patients with silent claudication. Particular detail should be paid to flap thickness especially in patients with increased body habitus. Radiation therapy has on average 20% flap volume-reducing effect, and an overestimation of volume in patients who will be undergoing radiotherapy should lead to the best contour and cosmetic outcomes.

## 1. Introduction

The anterolateral thigh- (ALT-) free flap was first described in 1984 by Song et al. and has since gained popularity, particularly in head and neck reconstruction [1]. This fasciocutaneous flap, based on the descending branch of the lateral femoral circumflex artery, initially became popular in Asia but has also more recently become a frequently used flap in Western countries. One of the main reasons this flap was less popular in Western countries is because of the tendency for Western patients to have thicker skin when compared to their Asian counterparts and the variable diameter, location, and number of cutaneous perforators. The attributes that make this a versatile “workhorse” flap include the remote location from the potential defect with low donor-site morbidity, a long large-diameter pedicle, a large skin paddle, the absence of patient repositioning which also allows for a 2 team approach, and the availability of the lateral cutaneous nerve of the thigh to provide sensation. 

Still despite all that has been described about the ALT flap, there is plenty of room for research and innovation. The flap is not appropriate for all defects, and careful selections based on patient characteristics, defect size, and location are critical as in all reconstructive surgeries. 

This paper will summarize two recent ALT studies from our institution focusing on the utility of colour flow Doppler's ultrasonography and volumetric changes with radiation [2]. Lastly, we will discuss recommended changes to practice due to the aforementioned studies. 

## 2. Clinical Utility of Colour Flow Doppler's Ultrasonography

A number of authors have described the use of the handheld Doppler devices in pre-operative identification of the cutaneous perforators in the ALT [1, 3–8]. These perforators can either run between the rectus femoris and vastus lateralis muscle and traverse the fascia lata as septocutaneous perforators or they can traverse the vastus lateralis muscle and the deep fascia as musculocutaneous perforators to supply the skin. There is significant variability in this perforator anatomy, making for a more challenging flap elevation [4]. 

A study by Iida et al. assessing colour Doppler's flowmetry in preoperative assessment of perforators in 17 ALTs found that it had a 92% true positive rate and a 96% positive predictive value [9]. They suggest that this instrument increases the reliability and safety of elevating this flap [9]. Another study looking to assess the efficacy of handheld Doppler devices in the preoperative identification of the cutaneous perforators found that, despite being simple and convenient, they are not always accurate and should be used with caution [10]. In assessing 100 ALT-free flaps, they found great variability in the number (between 1 and 3) and location of the perforators. The perforator that was the most consistent was that located near the midpoint of the line between the anterior superior iliac spine (ASIS) and the lateral portion of the patella, as frequently described in the literature [3, 8, 10]. They also looked within a 3 cm radius 5 cm proximal and distal to this midpoint. They found a single perforator in 22% of cases, two in 54%, and three in 24% using this technique [10]. The midpoint perforator was the most consistent one being present almost 90% of the time [10]. The sensitivity and specificities of using such a device was 91–100% and 0–55%, respectively, suggesting significant inaccuracies, and; thus, we agree with their conclusion that the Doppler should only be used as an adjunct in the identification of the perforators but should not be solely relied on [10]. 

It is well known that Western patients have increased body habitus compared to their Asian counterparts. Along with this comes an increased risk of diabetes, hypercholesterolemia, and ultimately vascular disease. Also, several authors have documented unexpected intraoperative absence of suitable cutaneous perforators in up to 5% of cases [8, 11–13]. 

A study by Tsukino et al. compared the use of a handheld acoustic Doppler to the colour Doppler ultrasonography and found, not surprisingly, that colour Doppler's examination was significantly more accurate and had a 100% concordance rate with surgical findings. In contrast, they concluded that the acoustic Doppler examination was unreliably with a 40% concordance rate [14]. 

Although it is common practice to use the handheld acoustic Doppler device for pre-operative identification of the perforators very few have advocated for increased use of colour Doppler's ultrasonography. A recent study from our centre looked at the use of this device [2]. We performed this study for a number of reasons. Several authors have documented unexpected intraoperative absence of suitable cutaneous perforators in up to 5% of cases [8, 11–13]. Undiagnosed peripheral arterial disease (PAD), present in more than one-quarter of elderly patients, may contribute to this challenge as well as the fact that reliance on a history of symptoms of claudication misses the diagnosis of PAD in 90% of patients [15–17]. This undiagnosed PAD may lead to vascular compromise of the donor extremity and failure of the transfer due to diseased pedicle vessels. An additional benefit to the use of the Doppler ultrasound is the assessment and measurement of thigh thickness which will affect the suitability of this site for free tissue transfer. 

Our study found that the colour flow Doppler changed our management for a number of patients. It diagnosed two patients with bilateral silent infrainguinal claudication, and; thus, the ALT was not used for either of these patients [2]. Two other patients had the same finding but only ipsilateral to the primary defect, and; thus, the contralateral side was used in these patients [2]. The colour flow Doppler also underestimated flap thickness but was statistically related to the actual flap thickness which was helpful in our planning of the ALT harvest [2]. 

Since many of our head and neck oncology patients requiring free tissue transfers have significant risk factors for PAD, such as advanced age, smoking, hypercholesterolemia, diabetes, and hypertension, the use of the colour flow Doppler can be quite helpful in selecting and identifying those patients with silent claudication, avoiding the risk of flap failure and significant morbidity to the donor site, and allowing for other free tissue sites to be considered. 

Nonetheless, we do not routinely use the colour flow Doppler on all patients undergoing an ALT, but we consider the use of this device in all patients with significant risk factors especially in those with no symptoms in the leg. Due to its predictive nature, it is also helpful in assessing those patents in which flap thickness is a concern. 

## 3. ALT for Parotidectomy Defects: Contouring, Volume, and Changes with Radiotherapy

Flap thickness and volume are critical when trying to fill defects such as that after parotidectomy. Facial contour is affected by flap volume and thickness and has important cosmetic effects, thereby, affecting patients' quality of life. A quality of life study by Lutz and Wei showed that more than half of the 125 postparotidectomy patients were dissatisfied with their facial contour [18]. 

We prefer using a free flap to fill parotidectomy defects as it provides relatively accurate initial tissue volume transfer, stability of facial contour, reduction of radiation-induced complications, and ability to perform adjunctive facial reanimation. A study evaluating cosmetic and symmetry outcomes of patients undergoing free flap reconstruction (with either radial forearm or ALT) when compared to those that did not found that those with free flaps tended to perceive better facial symmetry. Reconstructed patients had a 5% increase in volume compared to the nonoperated side [19]. Conversely, nonreconstructed patients had a 12% volume loss compared to the nonoperated side [19]. They conclude that free flap reconstruction for postparotidectomy defects is a good option for patients in whom facial volume asymmetry is a concern [19]. 

We have, however, noticed that after radiation there is a substantial flap volume reduction. For this reason, we have recently undertaken a prospective study that has been submitted but has yet to be published, assessing flap volume changes with radiation [20]. In brief, we assessed intra-operatively, using a modified Archimedes' principle, defect size as compared to flap size and found that our flaps were of approximately 10% greater volume [20]. We also assessed pre-compared to 3 months postradiation treatment CT scan flap volume and found a 20% reduction [20]. 

This has very important implications for flap design, as the reconstructive surgeon should slightly overestimate the volume, by approximately 20% in patients that will be undergoing postoperative external beam radiation therapy. This should lead to the optimal facial contour leading to more symmetrical facial appearance.

## 4. Conclusion

The ALT has gained popularity in Western countries but is not appropriate for all defects. Careful selection based on patient characteristics and defect size is critical as in all reconstructive surgeries. Special care should be made to identify patients at high risk for PAD, and if the reconstructive surgeon has any concerns, pre-operative colour flow Doppler's ultrasonography should be undertaken to better characterize the perforator anatomy and avoid inappropriate flap elevation in patients with silent claudication. Particular detail should be paid to flap thickness especially in patients with increased body habitus. Radiation therapy has a flap volume-reducing effect, and an overestimation of volume in patients who will be undergoing radiotherapy should lead to the best contour and cosmetic outcomes.
